# The complete chloroplast genome of *Osteospermum ecklonis* (DC.) Norl. (Asteraceae: Asterodae: Calenduleae), an ornamental plant

**DOI:** 10.1080/23802359.2022.2101394

**Published:** 2022-07-28

**Authors:** Jianhua Yue, Yan Dong, Shoufu Gong

**Affiliations:** a School of Horticulture, Xinyang Agriculture and Forestry University, Xinyang, Henan, PR China; b School of Forestry, Xinyang Agriculture and Forestry University, Xinyang, Henan, PR China

**Keywords:** *Osteospermum ecklonis* (DC.) Norl., chloroplast genome, phylogeny, Asteraceae

## Abstract

*Osteospermum ecklonis* (DC.) Norl. 1838 is a herbaceous and perennial plant native to South Africa. It is an ornamental plant that shows great commercial potential. In the present study, the complete chloroplast (cp) genome was 151,295 bp in total length, and 127 genes were identified, including 85 protein-coding, 34 tRNA, and eight rRNA genes. The cp genome includes a large single-copy (LSC) region of 83,293 bp, a small single-copy (SSC) region of 18,012 bp, and a pair of inverted repeats (IRs) regions of 24,995 bp. The phylogenetic relationship of *O. ecklonis* revealed by cp genome provides a foundation for future studies of the phylogeny in the Asteraceae.

The genus *Osteospermum* (family Asteraceae) contains about 70 species (Jakupovic et al. 1988), many of which are used to study plant modeling, flower form, and colors (Seitz et al. 2007). One of these species, *Osteospermum ecklonis* (DC.) Norl. 1838 is a herbaceous, perennial plant native to South Africa (Faccioli et al. 2000; Vaira et al. 2000; Laura et al. 2010). This species is commonly used for potted plant, landscaping and floriculture worldwide (Vaira et al. 2000; Laura et al. 2010).

Asteroideae is the largest subfamily of the Asteraceae family, furthermore, the widespread phenomenon of hybridization in the Asteraceae was investigated (Liu et al. 2015). Therefore, there has been considerable debate over the systematic position of some genera in the Asteraceae, including the genus *Osteospermum* (Barker et al. 2009). The chloroplast (cp) genome has been used to investigate the phylogenetic information of plants because of its maternal inheritance and conserved structure (Wang et al. 2018). In this present study, the complete cp genome of *O. ecklonis* was assembled and analyzed to better understand the phylogenetic position of *O. ecklonis*.

*O. ecklonis* leaf specimens were sampled from Xinyang Baihua Garden, Henan Province, China (114° 09′ E, 32° 14′ N) and preserved in liquid nitrogen. Afterwards, these specimens (Bio-sample accession: SAMN25210006) were stored in the −80 °C refrigerator of the Horticultural Plant Biotechnology Laboratory, Xinyang Agriculture and Forestry University. A specimen was deposited at the Herbarium of the Horticultural Plant Biotechnology Laboratory, Xinyang Agriculture and Forestry University (Contact person: Jianhua Yue, jhyues@163.com) under the voucher code XOE2201107. DNA was extracted from leaf tissues by the CTAB method (Odahara et al. 2009), after which the DNA sample was sent to Shanghai Origingene Biotechnology Co., Ltd. (Shanghai, China) to construct a DNA library and was sequenced by using the Illumina NovaSeq 6000 sequencing platform (San Diego, CA). Approximately, 12.6 GB of raw data was generated with 150 bp paired-end read lengths. The data were filtered by using NOVOPlasty (Dierckxsens et al. 2016), and the reads were then viewed and edited by using Bandage (Wick et al. 2015). The cp genome annotation was performed by Geneious v 11.1.5 (Biomatters, Auckland, New Zealand) (Kearse et al. 2012).

The complete cp genome of *O. ecklonis* has a typical conserved quadripartite structure of 151,295 bp in length with an overall GC content of 37.66%, containing four distinct regions: a large single-copy (LSC) region (83,293 bp), a small single-copy (SSC) region (18,012 bp), and a pair of inverted repeat (IR) regions (24,995). The complete cp genome consisted of 127 genes, including 85 protein-coding, 34 tRNA, and eight rRNA genes.

Then, 36 cp genome sequences were selected for the phylogenetic analysis to investigate the phylogenetic relationship of *O. ecklonis*, and all of the sequences were downloaded from NCBI GenBank. The sequences were aligned via MAFFT v7.307 (Katoh and Standley 2013), and the phylogenetic tree was constructed by using MEGA X (Kumar et al. 2018). The robustness of the topology was calculated using the maximum likelihood method with 1000 bootstrap replicates. The phylogenetic tree revealed that the genus *Osteospermum* was placed in the Calenduleae (Asterodae) of the Asteraceae family (Figure 1). Furthermore, the phylogenetic information suggested that the supertribe Senecionodae should be merged into the supertribe Asterodae. The result was highly consistent with the phylogenetic relationships of the *Osteospermum* species by using the low-copy nuclear gene methods (Liu et al. 2015). This study provides a foundation for future studies of the phylogeny in the Asteraceae.

**Figure 1.**
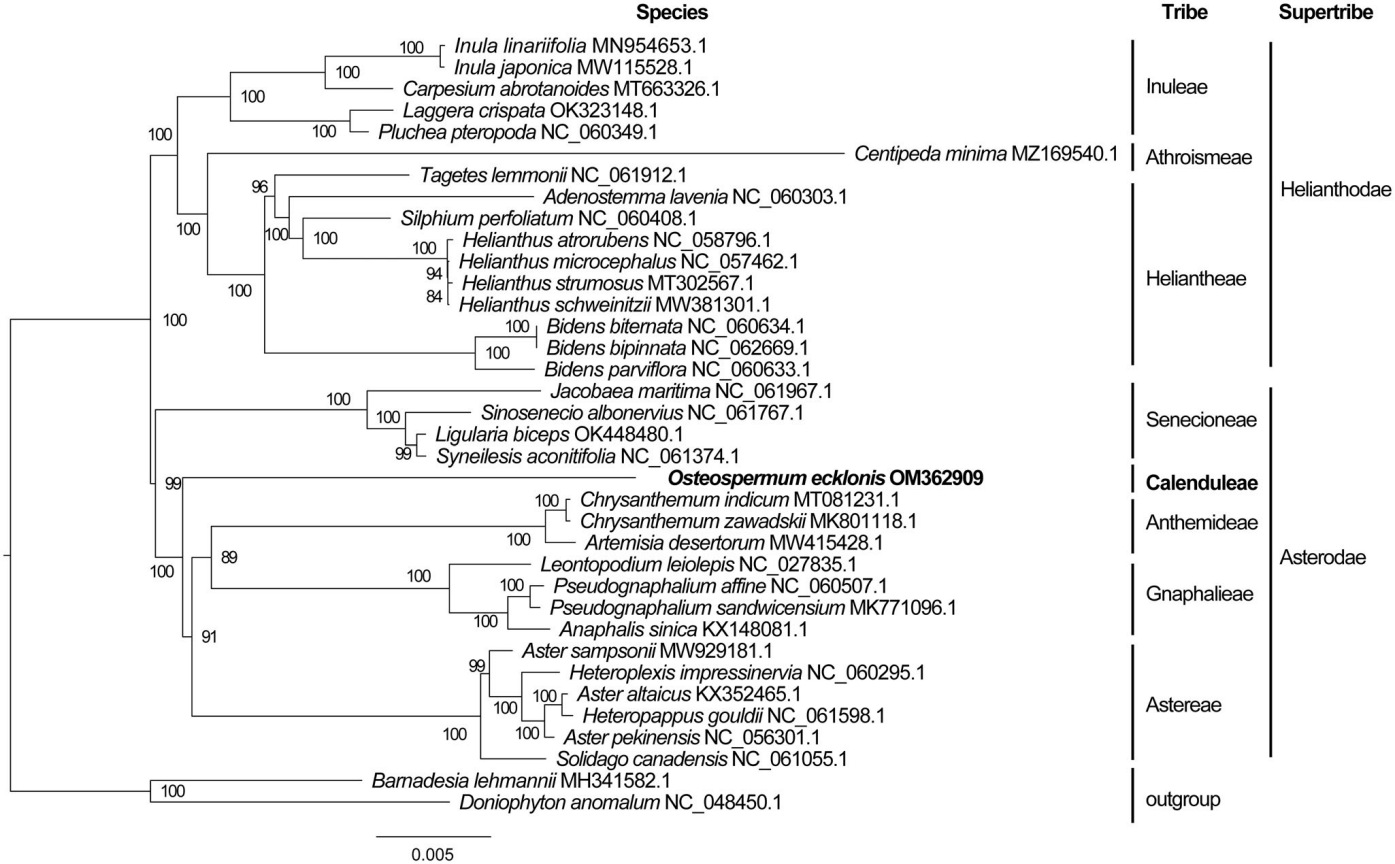
Maximum-likelihood phylogenetic tree based on complete cp genomes. Numbers close to each node are bootstrap support values.

## Data Availability

The data that support the findings of this study are openly available in GenBank of NCBI at https://www.ncbi.nlm.nih.gov/. The complete cp genome has been deposited in GenBank under the accession no. OM362909. The associated Bio-project, SRA, Bio-sample numbers are PRJNA799816, SRX13877376, and SAMN25210006, respectively.
